# Editorial: Common and distinct mechanisms of migraine and stroke

**DOI:** 10.3389/fncel.2023.1171836

**Published:** 2023-03-13

**Authors:** Rashid Giniatullin, Roustem Khazipov, Arn M. J. M. van den Maagdenberg, Jukka Jolkkonen

**Affiliations:** ^1^A.I.Virtanen Institute for Molecular Sciences, University of Eastern Finland, Kuopio, Finland; ^2^INMED, INSERM UMR1249, Aix-Marseille University, Marseille, France; ^3^Laboratory of Neurobiology, Kazan University, Kazan, Russia; ^4^Department of Human Genetics, Leiden University Medical Center, Leiden, Netherlands; ^5^Department of Neurology, Leiden University Medical Center, Leiden, Netherlands

**Keywords:** brain ischemia, migraine, spreading depolarization (SD), peri-infarct depolarizations, stroke

The Research Topic “*Common and distinct mechanisms of migraine and stroke*” is focused on the underlying mechanisms of both disorders. Both migraine and stroke are common and costly disorders that pose a great burden on the patient, their family, and society. Epidemiological studies have demonstrated a bidirectional comorbidity, which poses the question to what extent similar molecular mechanisms lead to the disorders and the co-occurrence in a patient. Evidence for a mechanistic link between stroke and migraine is strongest for migraine with aura and suggests that spreading depolarization (SD) in the brain is a key shared mechanism. Still, other mechanisms, e.g., neuroinflammation and oxidative stress, likely contribute to both pathologies, and a hypercoagulable state may also play a role in both migraine and stroke (Figure 1). Whereas, current evidence suggests a “stroke-migraine SD continuum,” we lack detailed knowledge on the full spectrum of underlying neurobiological mechanisms in migraine and stroke, hence the focus of the Research Topic.

**Figure 1:**
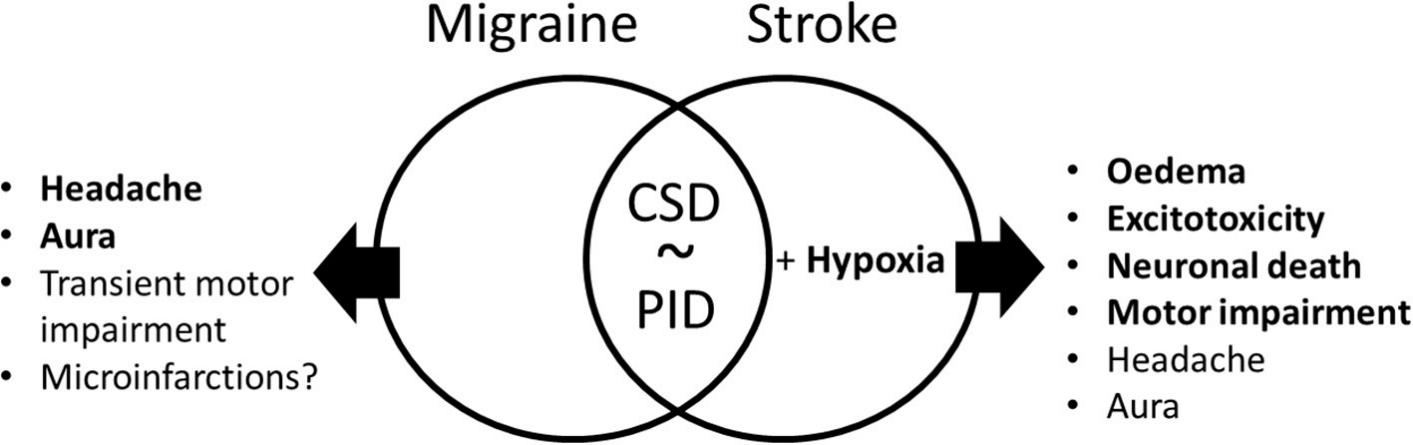
Common and distinct symptoms and mechanisms in migraine and stroke. Both migraine and stroke might have common symptoms, such as headache and aura, as well as sustained in stroke and transient in familial hemiplegic migraine motor impairment. The central mechanism in stroke is neuronal death, while there is some evidence of the presence of microinfarctions in migraine. The best obvious common mechanism of the two disorders is cortical spreading depolarization (CSD) in migraine with aura that is similar to peri-infarct depolarization in ischemic stroke. The PID in stroke is also associated with local hypoxia, which exaggerates the outcome of this pathology via edema, excitotoxicity, and finally leads to neuronal death.

The presented research aims at elucidating disease mechanisms that play a role in stroke and, to a certain extent, shed light on the relationship between stroke and migraine (Figure 1). The collection of contributions on SD is crowned by a comprehensive review by Lemale et al. on the SD continuum in various clinical conditions, ranging from migraine to ischemic stroke and traumatic brain injury. A claim was made that SD represents a prime, most neglected, pathophysiological process in acute neurology. The authors review the basal mechanisms underlying SD and associated changes in cortical activity and provide evidence that associations between SD and spreading depression of activity are by no means trivial, but rather pose unsolved mechanistic puzzles. Lu and Scharfman presented a methodological advance to study SD using hippocampal slices in a submerged chamber with a flow of low-magnesium/high-potassium solution above and below the slice that reliably evokes recurrent SDs and seizure-like events. Their model provides new opportunities to explore SDs and interactions between SDs and paroxysmal activities. Gainetdinov et al. presented a quite unique developmentally immature SD phenotype with small depolarizations, transient recovery, and maintained excitability in neonatal cortical neurons during SD evoked by oxygen-glucose deprivation.

In search for a treatment, Wei et al. explored for beneficial effects of oxymatrine, a quinolizidine alkaloid used in traditional Chinese herb medicine, against neonatal hypoxic–ischemic brain damage. Oxymatrine exerted neuroprotective effects both *in vivo* and *in vitro* in models of hypoxia-ischemia that involve the PI3K/Akt/mTOR pathway and attenuation of excessive autophagy.

Suleimanova et al. investigated through computational modeling, nociceptive signaling in familial hemiplegic migraine type 3 (FHM3) caused by gain-of-function mutations in the *SCN1A* gene. The gene encodes the α_1_ subunit of voltage-gated Na_V_1.1 sodium channels with mutations linked to epilepsy and stroke, which are comorbid with migraine. The combined activation by ATP and serotonin of purinergic P2X3 and serotonergic 5-HT3 receptors, expressed on trigeminal afferents, resulted in high-frequency spiking activity in the disease condition. Of several FHM3 mutations, missense mutation L263V has the longer activation state and induced the most profound spiking activity in the meninges, predicting more severe headache symptoms in patients with this mutation.

Linstra et al. investigated the connection between migraine and stroke, and especially sex differences in the risk of stroke in patients with migraine, among 2,492 patients with ischemic stroke, of which 425 also had migraine. A history of migraine appears not to be associated with changes in the prevalence of conventional cardiovascular risk factors. However, young women with migraine had a higher risk for stroke whereas men with migraine had a poorer outcome compared to those without migraine.

van der Weerd et al. performed a systematic review of 24 studies with data on sex differences in hemostatic factors in ischemic stroke. Data from 25 factors were investigated in a total of 7,247 patients. Although lack of data, foremost of replication of findings in independent studies that calls for follow-up studies, current data suggested higher levels of procoagulant factors (FVII-C, FXI, D-dimer, tPA, and PAI-1) in women and higher coagulant inhibitors (protein-S and P-selectin) in men.

In a mechanistic study, Liu Y. et al. explored the DNA methylation status in blood samples of stroke patients. Compared to controls, almost 300 genes showed different methylation levels, of which seven were validated in larger patient groups, with *CAMTA1* being significantly altered. The authors also generated a *CAMTA1* knockout in SH-SY5Y neuroblastoma cells, revealing an enrichment of gene sets that are involved in cellular proliferation and cell cycle. When knockout cells were subjected to oxygen-glucose deprivation and reperfusion, which induces neuronal injury related to brain ischemia, it was shown that cyclin D1, an essential regulator of cell cycle progression, was upregulated, suggesting that *CAMTA1* plays a role in stroke.

The last contribution to the Research Topic came from Liu C. et al. who used a bioinformatics approach to identify possible therapeutic targets related to ferroptosis, a type of programmed cell death characterized by iron-dependent accumulation of lipid peroxides. By combing ischemic stroke microarray data with publically available data on ferroptosis regulators and disease associations, they identified 33 differentially expressed genes relevant to ferroptosis. Four of which, *HMOX1, STAT3, CYBB*, and *TLR4*, were enriched in the HIF-1 signaling pathway. Subsequent analyses revealed an upregulation of these genes in mouse brain tissue after ischemic stroke by middle cerebral artery occlusion (MCAO)/reperfusion. Dexmedetomidine, a selective α2-receptor agonist, reduced MCAO-induced cell death and improved neurobehavioral deficits. Also, it reversed the abovementioned expression changes as well as levels of inflammatory factors TNFα and IL-6, thereby providing evidence that the compound inhibits ferroptosis mechanisms in ischemic stroke.

In summary, this Research Topic highlights common mechanisms and complex, often intertwined, relationships between migraine and stroke. Newly discovered mechanisms revealed potential therapeutic targets that need confirmation in clinically predictive animal models. Large patient cohort studies revealed clinical risk factors and sex differences in migraine and stroke. Together, the collected articles are an important reference to guide the field in finding fundamental answers to the overlapping pathology in migraine and stroke, which eventually is expected to lead to safe, effective, and personalized treatments.

## Author contributions

All authors listed have made a substantial, direct, and intellectual contribution to the work and approved it for publication.

